# Evaluation of the “safe multidisciplinary app-assisted remote patient-self-testing (SMART) model” for warfarin home management during the COVID-19 pandemic: study protocol of a multi-center randomized controlled trial

**DOI:** 10.1186/s12913-021-06882-7

**Published:** 2021-08-26

**Authors:** Lei Chen, Yang-Zhao Zhou, Xin-Min Zhou, Li-Ming Liu, Ping Xu, Xia Zhang, Sheng-Lan Tan

**Affiliations:** 1 Department of Pharmacy, The Second Xiangya Hospital, Central South University, Changsha, 410011 China; 2 Institute of Clinical Pharmacy, The Second Xiangya Hospital, Central South University, Changsha, 410011 China; 3 Department of Cardiovascular Surgery, The Second Xiangya Hospital, Central South University, Changsha, 410011 China

**Keywords:** Warfarin, Patient self-testing, Safety, Effectiveness, Cost-effectiveness

## Abstract

**Background:**

Warfarin treatment requires frequent monitoring of INR (international normalized ratio) to adjust dosage in a therapeutic range. In China, patients living in small towns usually go to tertiary hospitals to get warfarin monitoring and dosing, resulting in low frequencies of follow-ups and high incidence of complications. Influenced by the COVID-19 pandemic, patients on warfarin have further reduced their visits to healthcare institutions. While patient self-testing (PST) via using a point-of-care testing device for INR measuring at home has been widely used in developed countries and demonstrated improved clinical outcomes compared to usual care in clinics, it is rarely applied in developing countries, including China. This proposed study will develop and assess the “Safe Multidisciplinary App-assisted Remote patient-self-Testing (SMART) model” for warfarin home management in China during the COVID-19 pandemic.

**Methods:**

This is a multi-center randomized controlled trial. We will carry out the study in three county hospitals, three small tertiary hospitals and three large tertiary hospitals with anticoagulation clinics in Hunan province of China. Eligible patients will be randomly assigned to the SMART model group (*n* = 360) or the control group (usual care clinic group, *n* = 360; anticoagulation clinic group, *n* = 120). Patients in the SMART model group do PST at home once every two to 4 weeks. Controls receive usual care in the clinics. All the patients will be followed up through outpatient clinics, phone call or online interviews at the 3rd, 6th, 9th and 12th month. The percentage of time in therapeutic range (TTR), incidence of warfarin associated major bleeding and thromboembolic events and costs will be compared between the SMART model group and control groups.

**Discussion:**

Patients in the SMART model group would show improved TTR, lower incidence of complications and better quality of life compared to the control groups. Our design, implementation and usage of the SMART model will provide experience and evidence in developing a novel model for chronic disease management to solve the problem of healthcare service maldistribution, an issue particularly obvious in developing countries during the COVID-19 pandemic.

**Trial registration:**

ChiCTR, ChiCTR 2000038984. Registered 11 October, 2020.

## Background

In line with the increasing aging population, the incidence and prevalence of atrial fibrillation (AF) and other thromboembolic diseases have dramatically increased and become a significant health problem worldwide [1]. Patients with these conditions should take long-term or lifelong anticoagulant drugs to prevent or treat thrombosis. It’s estimated that at least 0.6% of people in China need anticoagulation therapy [2, 3].

Although the new oral anticoagulants (NOACs) have been used as alternatives to warfarin due to good safety and less monitoring, warfarin remains the first line anticoagulant in certain conditions, including mechanical heart valve replacement, severe mitral stenosis, renal failure and etc. In addition, anticoagulant choice is impacted by patients’ individual preference, insurance allowance and personal economic situation. Therefore, warfarin will continue to be widely used across the world.

Warfarin treatment is cheap and effective, but requires frequent monitoring to adjust dosage under professional guidance. In China, patients and families from rural locations often need to travel several hours to tertiary hospitals in big cities to get quality warfarin monitoring and dosing, resulting in poor compliance, limited monitoring frequency, high incidence of warfarin related complications and increased costs. During the COVID-19 pandemic, many patients with chronic diseases, including patients on warfarin, have reduced their visits to hospitals due to the social distancing policy and shortage of medical resources.

In China, the percentage of time in therapeutic range (TTR, a tool of measuring warfarin treatment effects) was less than 40%, which was significantly lower than that of 62% in western countries [4]. Clinical observation studies found that warfarin associated ischemic stroke and intracranial bleeding were respectively 5.23 and 2.94% in patients from China, significantly higher than patients from the rest of the world (2.94 and 0.63%, respectively) [5]. In warfarin-treated patients in China, the median in-hospital direct cost per patient was about $3, 000 for thrombosis and $4, 500 for intracranial hemorrhage [6]. These data suggest that warfarin management in China needs further improvement. An investigation study launched by the PI using the Andersen’s Health Services Utilization Behavioral Model in 469 patients on warfarin indicated that long time and high cost for travel, low family income, low quality of warfarin monitoring and dosing in local hospitals were dominant reasons for missing follow-ups (data not published yet).

Anticoagulation clinics (AC) have been proved safer and more effective than usual care in warfarin management [7, 8], but there are only about 50 ACs located in big cities of China (mainly run in a physician-pharmacist cooperative AC pattern) at present. The number of ACs is too low, and it is not easy to open more ACs due to limitation of health care professionals and longtime education.

Point-of-care testing (POCT) device designed for measuring INR by patients or their families was first developed in 1980s in America [9] and then widely used in many developed countries. Compared to traditional laboratory INR test in outpatient clinics, POCT has great advantages in convenient home testing and fast result reporting. Soon patient self-testing (PST) for warfarin home management by using POCT devices and telehealth has been rapidly applied in developed countries. Although the PST model of warfarin management has demonstrated improvement of clinical outcomes compared to usual care clinics and anticoagulation clinics [10], it is rarely applied in developing countries, including China.

The COVID-19 pandemic has motivated significant changes to health-care system, many of which will have a lasting impact. Telehealth or telemedicine, which is defined as the use of medical information over a spatial distance through electronic communication to improve a patient’s health, has been widely implemented during and after the COVID-19 pandemic all over the world [11, 12]. In the early stage of the pandemic, we had integrated telehealth and INR testing POCT device to guide a few patients to monitor INR and adjust warfarin dosage at home. Those patients were managed very well with improved TTR and satisfaction.

In order to improve warfarin management in China during the COVID-19 pandemic, we have proposed a novel management mode called the “Safe Multidisciplinary App-assisted Remote patient-self-Testing (SMART) model” for warfarin home management. Our team has developed a mobile phone based app which called “XY” app, (XY is abbreviated for Xiangya Second hospital) to efficiently assist managing warfarin with different strategies based on patients’ risks of bleeding or thrombosis. Moreover, we will use the resources of medical alliance (of which tertiary hospitals collaborate with local hospitals for patient treatment and referral) to train local health care providers and establish multidisciplinary teams to manage patients together.

### Aim

This study aims to improve patients’ medical outcomes and quality of life, reducing patients’ burden of frequent follow ups in tertiary hospitals, and decreasing patients’ dependence on health care professionals to a certain extent by implementing the SMART model to manage patients on warfarin during the COVID-19 pandemic. Moreover, our study will provide experiences of telehealth implementation during and after the COVID-19 pandemic, which would be particularly helpful for areas with uneven medical quality. Ultimately, we expect to expand the SMART model to manage other chronic diseases.

## Methods/design

### Study design

**Fig. 1:**
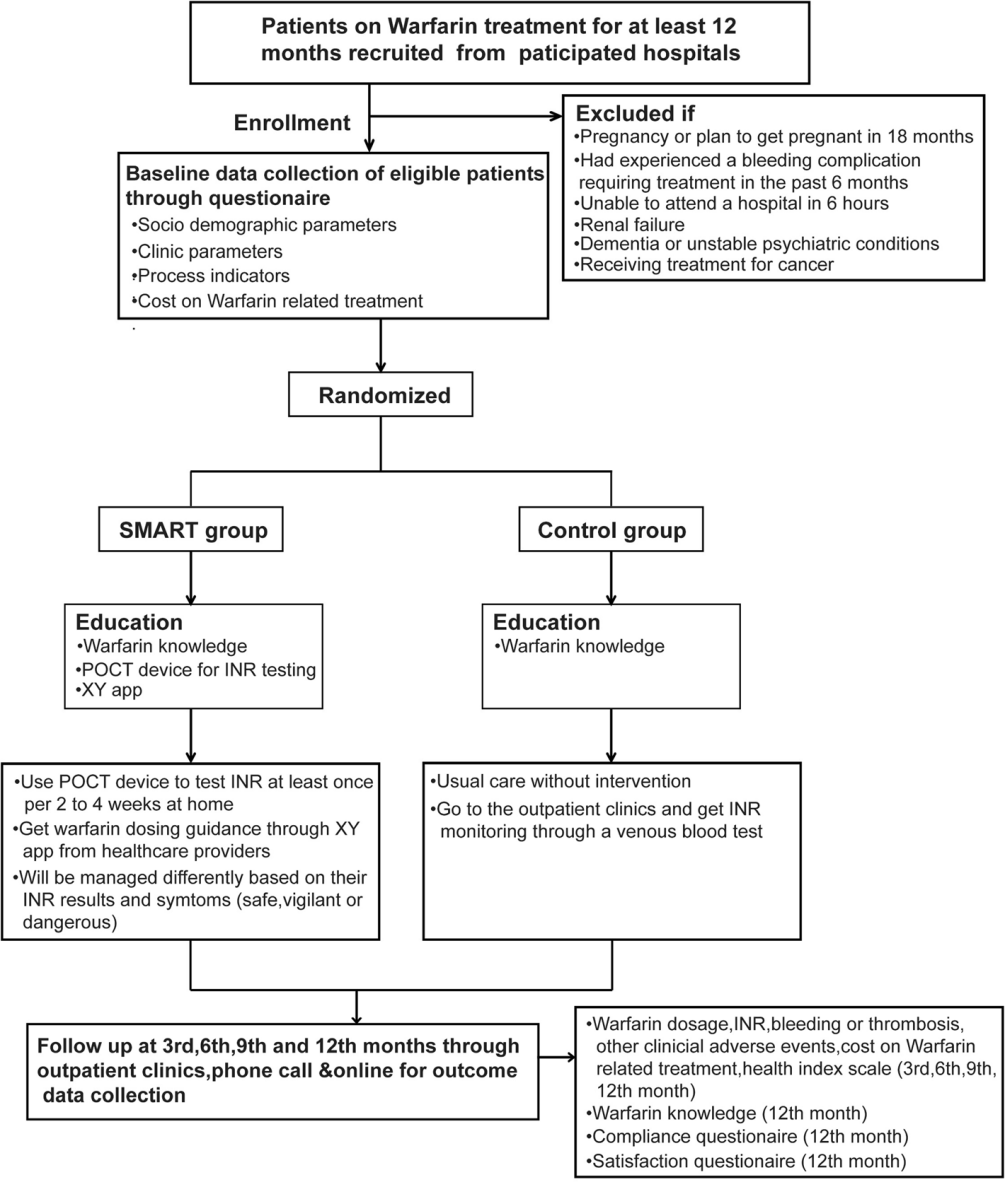
Flow chart of the study

This is a multi-center randomized controlled trial. We will carry out the study in three large tertiary hospitals with anticoagulation clinics, three small tertiary hospitals and three county hospitals in Hunan province of China. Eligible patients will be randomly assigned to the SMART model group or the control group (usual care clinic and anticoagulation clinic). Patients in the SMART model group do PST at home once every two to 4 weeks. Control patients receive usual care without intervention. All the patients will be followed up through outpatient clinics, phone call or online interviews at the 3rd, 6th, 9th and 12th month. The percentage of time in therapeutic range (TTR), incidence of warfarin associated major bleeding and thromboembolic events and costs will be compared between the SMART model group and control groups. Figure 1 describes the study design.

### Study participants and inclusion criteria

Patients eligible for this project have to comply with all of the requirements below: are expected to be on warfarin treatment for the duration of at least 12 months, accept the study protocol and randomization, and sign an informed consent. Patients who fall into any of the following criteria are excluded in this project, including pregnancy or plan to get pregnant in 18 months, had experienced a bleeding complication requiring treatment in the past 6 months, are unable to attend a hospital in 6 hours, renal failure (hemodialysis or glomerular filtration rate < 10 mL/min), dementia or unstable psychiatric conditions or receiving treatment for cancer.

A patient who has been enrolled in the project will be considered as a dropout if meets any of the following criteria and the reason will be recorded: the patient decides to withdraw for any reason; the patient in the SMART model group does not follow the health-care professionals’ instruction for at least twice; the researcher considers the patient is no longer physically and/or psychologically fit to remain in the study. The impact of dropouts on statistical analyses, including intention to treat, will be used by carrying the last observation forward.

### Study settings

As the quality of medical care in China is unbalanced distributed, we will carry out the study in hospitals respectively representing low (county hospitals), medium (small tertiary hospitals) and high levels (big tertiary hospitals with ACs) of anticoagulation management in Hunan province to comprehensively evaluate the SMART model. Three hospitals of each level are randomly selected, thus finally nine hospitals are included in this study. Our hypothesis is that the SMART model is not only better than the usual care (UC, physician managed clinics) and the AC management, but also could be applied in different levels of hospitals in China.

### Procedures

#### Training health care providers

All the participated health-care providers from nine hospitals will be organized together to receive both face-to-face seminar training courses and online meetings to master the study protocol and the measurements of managing patients. Table 1 shows the detailed training courses. In each hospital, a multidisciplinary team consisting of a physician and a pharmacist will manage their patients in the SMART model group together. For the three county hospitals, experienced pharmacists who manage the anticoagulation clinics in the tertiary hospitals will also join their multidisciplinary team to help manage patients with the aim of assuring patients’ safety.

**Table 1:** Training courses for health care providers from participated hospitals

Training courses	Details
**Warfarin management**	1.Theoretical knowledge ① Basic knowledge of warfarin ② The standard protocol of warfarin management with patient self-testing ③ Warfarin monitoring and dosing at different situations ④ Adverse events treatment, including thrombosis, minor bleeding and major bleeding ⑤ Introduction of new oral anticoagulants ⑥ Laboratory tests interpretation ⑦ Patient education on warfarin, including medication adherence, drug/food interactions, diet, exercise and mental health 2. Practice in simulate patients 3. Tests to assess participant’s theoretical knowledge and practice skills
**Related-disease management**	1.Theoretical knowledge ① Atrial fibrillation ② Valvular heart disease ③ Deep venous thrombosis and pulmonary embolism ④ Stroke ⑤ coronary heart disease 2. Practice in simulate patients 3. Tests to assess participant’s theoretical knowledge and practice skills
**SMART Model**	1.Theoretical knowledge ① Usage of the POCT device ② Usage of the “XY” app ③ Stratified management based on patient risks ④ Communication skills with patients via the “XY” app ⑤ Communication between local hospitals and tertiary hospitals ⑥ Patients’ referral 2.Practice in simulate patients 3. Tests to assess participant’s theoretical knowledge and practice skills

#### Enrolling patients

Patients on warfarin attending the participated hospitals will be invited to join in the study. If the patients show interest, then the design and rationale of the study will be explained. The research staff will identify which patients are potentially suitable candidates for the study. After the eligible patients agree and sign the informed consents, the patients will be randomly assigned to either the SMART model group or the control group according to the protocol.

#### Baseline interview

All the participated patients will be required to complete a survey either via an online survey through their mobile phone or via a printed brochure. The contents of the survey contain three parts, including patients’ clinical parameters (warfarin indication, start date of warfarin, warfarin dosage, previous TTR, history of thrombosis or major bleeding, concomitant diseases and medicine), behaviour indicators (warfarin knowledge, anticoagulant compliance, treatment behavior and quality of life) and costs on warfarin related treatment.

#### Randomization

**Fig. 2:**
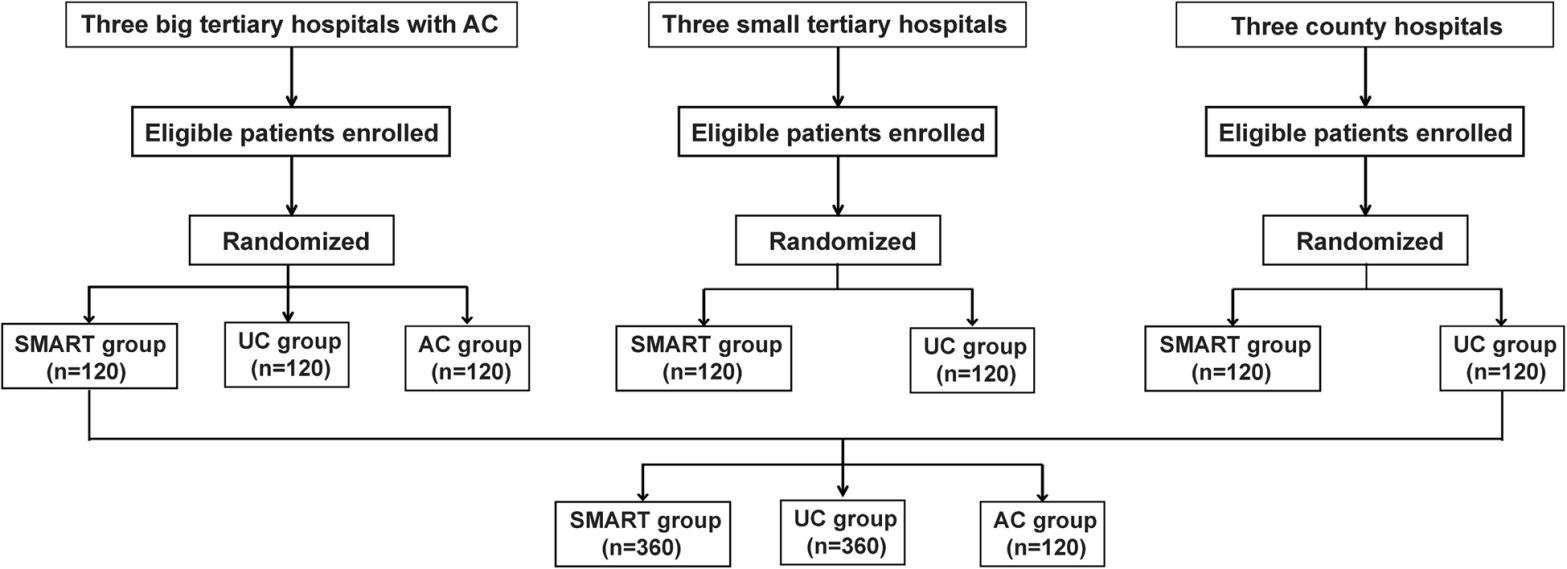
Flow chart of patients’ grouping and allocation in different levels of hospitals. AC: anticoagulation clinic; UC: usual care clinic; SMART: the “Safe Multidisciplinary App-assisted Remote patient-self-Testing” model

In low and medium level hospitals, only the UC group is used as the control group, thus eligible patients on warfarin will be randomly assigned to either the SMART model group or the UC group. While in the high level hospitals, both AC and UC are used as the control groups, thus eligible patients will be randomly allocated to the SMART model group, the AC group or the UC group. For each level of the three hospitals, a total of 120 patients in each arm will be enrolled. The flow chart of patients’ grouping and allocation is shown in Fig. 2. Patients’ randomization will be carried out by research staff using a standard protocol available in the website (http://stattrek.com/statistics/random-number-generator.aspx).

#### Educating and training patients

All the enrolled patients will attend a warfarin education session by the research team after they sign the informed contents. The educational courses will include the mechanism of action of warfarin, blood tests of INR and target ranges, follow-up frequencies, potential signs and symptoms of over-coagulation and under-anticoagulation, drug/food interactions with warfarin, lifestyle, missed doses, over doses, adverse events treatment, etc. All the above-mentioned knowledge will be printed out and given to patients for home study. For patients allocated to the SMART model group, they will further attend a training program in groups of less than three people. They will learn how to use the POCT device, as well as report their INR results and receive instruction of warfarin dosage and follow-up dates through the XY app. Later, patients are required to use the POCT device to perform at least two reproducible INR measurements under the research staff’s supervision. These results will be compared with the laboratory INR measurement from a venous blood sample. If there are wide differences (> 0.3 INR units) between the POCT and laboratory INR tests, patients will be excluded from the study [13]. Patients or their guardians who fail to use the XY app to report data will also be excluded from the study.

#### Intervention protocol

As a general rule, patients in the SMART group are required to measure their INR once every two to 4 weeks by using the POCT device at home. However, if their INR is lower or higher than the target range, and the patient is advised to adjust the warfarin dosage, the patient will be required to do the POCT INR test again in a few days. Table 2 shows the stratified warfarin management method for patients in the SMART group. If a patient reports a therapeutic or only slightly out-of-range INR with no other issues, they will be labeled “safe” and receive instant feedback from the system regarding their warfarin dose and the next testing dates. A patient whose INR is higher than the target range but less than 4.5 without bleeding, or INR is less than the target range without thrombosis is labeled “vigilant”. He/she will be called by a health-care professional for detailed inquiry and will be advised to adjust warfarin dose or go to hospital. Any patient whose INR is higher than 4.5 or reports a symptom suggestive of major bleeding or thrombosis is labeled “dangerous”, and he/she should go to the local hospital or the tertiary hospital as soon as possible. This hierarchical management pattern can ensure the safety of the enrolled patients.

**Table 2:** Stratified warfarin management method in the SMART group

Label	Definition	Management
**Safe**	Patients have to meet all the conditions below: 1. INR is in the target range 2. Patients do not have bleeding, thrombosis or other symptoms 3. Patients do not require to take a new medicine recently 4. Patients are not diagnosed with a new disease recently	Health care providers will send a message like “please continue the current dosage of warfarin and check your INR on a schedualed date” through the “XY” app .
**Vigilant**	Patients have to meet one of the conditions below: 1. INR is ≥1.5 but lower than the target INR range 2. INR is higher than target range but ≤4.5 3. Patients have minor bleeding 4. Patients require to take a new medicine recently 5. Patients are diagnosed with a new disease recently 6. Patients have other minor symptoms, such as dizzy, weak, foot edema	Health care providers will call the patient in 8 h and give instructions with both oral guidance and app message. If necessary, some patients are advised to go to hospital for further treatment.
**Dangerous**	Patients have to meet one of the conditions below: 1. INR is < 1.5 or > 4.5 2. Patients have major bleeding 3. Patients have thrombosis 4. Patients have other severe symptoms, such as dyspnea, severe headache	The health care providers will call the patients immediately. Patients will be advised to go to the local hospital or tertiary hospital for further treatment based on their risks.

#### Control patients

Patients will receive the warfarin education courses the same as the SMART model group after they sign the informed contents as mentioned above. However, we will not intervene the warfarin manage mode in the control groups.

#### Follow-up visits and data collection

Each of the enrolled patients has to record his/her warfarin related information in a printed monitoring handbook, including INR results, warfarin dosage, other medicine, diet change, new disease, and thrombosis or bleeding events (if occurs). All the patients are required to receive follow-ups at the 3rd, 6th, 9th and 12th month through outpatient clinic visits, telephone or online interviews. At each follow-up time-point, the research stuff will collect the warfarin related information. Double data entry will be carried out to ensure data accuracy and data will be kept in encrypted mobile hard disks to protect patients’ privacy. Only the corresponding author has access to the final trial dataset. As shown in Table 3, patients are required to provide the information of warfarin dosage and INR results, the numbers of going to the outpatient clinics or inpatient treatment due to warfarin, quality of life as measured by the health index scale (EQ-5D-3L) [16], and the related treatment costs. At baseline and the last time visit, patients are required to fill in the Morisky anticoagulant compliance questionnaire [14] and the oral anticoagulation knowledge test [15]. An anticoagulation satisfaction survey by using the Duke Anticoagulation Satisfaction Scale [17] will be conducted to all the patients at the end of the study. The research staff will send text messages to remind the patients for follow-ups and all the participated patients will receive certain amount of compensation fees to sustain their involvement in the study.

**Table 3:** Measurement domains, survey methods and collection time-points

Variable	Component	Measurement methods	Baseline	3rd M	6th M	9th M	12th M
**Socio-demographic parameters**	Gender, age, telephone, address, education, occupation and annual income	Face-to-face interview	√				
**Clinical parameters**	Height, weight, warfarin indication, start date of warfarin, warfarin dosage, TTR calculated by INR tests in the preceding 12 months, history of thrombosis or major bleeding, concomitant diseases and medicine Warfarin dosage, INR, bleeding or thrombosis, other clinical adverse events	Face-to-face interview	√				
		Face-to-face interview, telephone or online interview		√	√	√	√
**Behaviour parameters**	Anticoagulant compliance, warfarin knowledge,	Morisky Anticoagulant Compliance Questionnaire [14], Oral Anticoagulation Knowledge (OAK) Test [15]	√				√
	Treatment behavior, quality of life	Self-made questionnaire, EQ-5D-3L Form [16]	√	√	√	√	√
	Anticoagulation satisfaction	Duke Anticoagulation Satisfaction Scale [17]					√
**Economic parameters**	Direct costs (INR test fees, outpatient clinic visit fees, hospitalization or emergency room visit fees if occurs), Indirect costs (deducted income due to absence of working, traffic and accommodation fees)	Face-to-face interview, telephone or online interview	√	√	√	√	√

### Outcomes

#### Primary outcome measure

The difference in TTR between the SMART model group and the control group during the 12 month period of study.

#### Secondary outcome measures

1. The numbers of INR results indicating excessive under-coagulation or over-anticoagulation (INR < 1.5 or INR > 5.0).
2. Serious adverse clinical events (clinical relevant non-major bleeding, major bleeding, thrombosis or death)
3. Patients’ quality of life.
4. Costs on warfarin management.

#### Data monitoring

As the corresponding author is the principle investigator and the Xiangya Second Hospital of Central South University is the coordinating center of the study, the Clinical Research Ethics Committee of Xiangya Second Hospital of Central South University has reviewed and approved this study protocol (Protocol version: XY2-POCT01). The committee monitors all human research conducted by stuff in the Xiangya Second Hospital, which is independent from the sponsor and competing interests. The three participated county hospitals do not have clinical research ethics committee, so they accept the ethical approval from Xiangya Second Hospital of Central South University. For the other five participated tertiary hospitals, their clinical research ethics committee will approve the study independently. We will seek approval from the clinical research ethics committee of the participated hospital for any important procedure modifications and inform patients according to their guidelines. We have registered the study in Chinese Clinical Trial Registry (No.ChiCTR2000038984). The research staff will report adverse events and other unintended effects of the trial intervention to the Ethics Committee and manage these events according to their guidelines.

#### Data analyses

We will report continuous variables as means ± SD and categorical variables as proportions. Data will be checked for normal distribution by means of the Kolmogorov–Smirnov’s test. In compliance with the distribution of data, Student’s t-test or Mann–Whitney’s U-test will be used for comparisons between the SMART model group and control group. Chi-square or Fisher’s exact tests will be used to compare between the SMART model group and control group with respect to categorical data. The control of anticoagulation will be assessed by evaluating TTR with the Rosendaal method [18] in each arm. The follow-up assessment data will be analyzed using Generalized Linear Mixed Model and survival analysis. *P* ≤ 0.05 is considered statistically significant. All tests are two tailed. From the perspective of health-care payer, we will conduct a cost-effectiveness analysis to assess the economic impact of the SMART model for warfarin home management. A Markov model, a half-cycle correction and a discount of 5% will be constructed or used to estimate the total costs and quality-adjusted life-years (QALYs) over the 2-year period (the outcomes beyond the observation period will be predicted according to the results obtained in the study). We will collect the direct and indirect costs of each enrolled patient on warfarin, evaluate their quality of life through EQ-5D-3L utility value from data provided by patients, calculate QALYs, and analyze the effects of multiple variables by the Linear Mixed Effects Model. The direct costs include laboratory INR test fees in the control groups (or POCT fees in the SMART model group), outpatient clinic visit fees (or supervision fees in the SMART model group), hospitalization fees and emergency room visit fees. The indirect costs include deducted income due to absence of working, traffic fees and accommodation fees. Because warfarin is very cheap, the medication fee of warfarin is not calculated in each group. The incremental cost effectiveness ratio (ICER) will be calculated according to the total costs and QALYs, and one-way and probabilistic analyses will be performed to verify the stability of the model.

### Sample size determination

In order to detect a hypothetical TTR difference of 5% between the SMART model group and the control group with a power of 80% and a significance of *P* = 0.05, a sample size of 31 patients in each group is required [19]. An additional 10% is added to allow for patients dropping out of the study, thus a sample size of at least 34 patients in each arm is needed.

### Dissemination

The research results will be disseminated through publications in peer-reviewed journals, academic conferences seminars and workshops. Articles and videos related to warfarin management made in plain words will be shared to public in website for free.

## Discussion

In a recent review, scientists believe SARS-CoV-2, the virus caused the Coronavirus Disease 2019 (COVID-19) pandemic, will continue to evolve and evade human immunity. They have predicted three scenarios, and the most likely scenario is that the COVID-19 pandemic will develop to an epidemic seasonal disease like influenza [20], which will influence human health for a long time. During the pandemic, many patients have delayed or avoided hospital visits due to fear of getting infected. This phenomenon is particularly obvious among the elderly patients or patients living in rural areas. Complying with the social distancing regulations, many countries are encouraging hospitals to conduct telehealth visits and other interactions with patients. As a result, the use of telehealth has been significantly increased in many countries, including China. Therefore, we believe COVID-19 will last for a long time and the telehealth will be widely accepted and applied in the healthcare system across the world.

While not new, warfarin home management via telehealth and a POCT device for INR measurement has received widespread acclaim in developed countries. There are mainly two patterns of warfarin home management. One pattern is patient self-testing, of which patients perform INR tests followed by the adjustment of warfarin dosage guided by a healthcare provider. The other pattern is patient self-management, of which patients perform INR tests and adjust warfarin dosage by themselves. Patients’ frequent home monitoring enables the abnormal INR values to be identified in time, attributing to lower risks of adverse events. A meta-analysis showed 50% reduction in the incidence of thromboembolic events in the warfarin home management group compared with the usual care group [10]. Warfarin home management has been proved as safe as traditional care in terms of major bleeding events [21]. The patients who received home management showed improved satisfaction and better quality of life compared to the usual care group [22]. Furthermore, warfarin home management is cost-effective [23]. As a result, coverage under Medicare was approved in 2002 for the indication of mechanical heart valves and in 2008 for other diseases including atrial fibrillation in the United States.

Nevertheless, the implementation of warfarin home management faces obstacles in developing countries, including legal and regulatory issues, patients lacking direct and timely access to professional recommendations from health care providers, lack of access to telehealth technology, and limited or no coverage of the POCT device from the government. The current proposed study will take several measurements (the SMART model) to ensure its implementation in China, including using a mobile phone app to assist caregivers managing warfarin more efficiently, establishing medical alliance and multidisciplinary teams to manage patients together to solve the problem of maldistribution of medical care, and using a hierarchical strategy to manage patients to assure their safety. As far as we know, this proposed study will be the biggest multi-center randomized controlled trial to investigate the safety, effectiveness and cost-effectiveness of warfarin home management in China. If our final data demonstrate the SMART model for warfarin home management is cost-effective, this pattern may be applied in more provinces and be expanded to other chronic diseases management.

In the proposed study, we will include hospitals with different qualities of medical service. On the one hand, as the current medical service level in the county hospitals is low, we expect that the differences in TTR, numbers of under-coagulation or over-anticoagulation, and serious adverse clinical events between the SMART model group and the control group may be significantly bigger than the other two levels of hospitals. On the other hand, patients’ quality of life and costs on warfarin management may be improved the most obviously in the big tertiary hospitals, as patients do not have to travel long way to the hospitals.

The biggest limitation of our proposed study is that the nature of the intervention method (the SMART model) excludes patients who are unable to use a POCT device correctly, or fail to report their INR results through a mobile phone based app.

In summary, we have developed a novel model (the SMART model) to investigate the safety, effectiveness and cost-effectiveness of warfarin home management in different areas of Hunan province in China. This model may be particularly useful to manage patients on warfarin during and after the COVID-19 pandemic.

### Trial status

Recruitment has begun in March 2021.

## Data Availability

The datasets generated and/or analyzed during the current study are not publicly available but are available from the corresponding author on reasonable request.

## Abbreviations

SMART: Safe Multidisciplinary App-assisted Remote patient-self-Testing
INR: International normalized ratio
TTR: Time in therapeutic range
AC: Anticoagulation clinic
POCT: Point-of-care testing
PST: Patient self-testing
UC: Usual care clinic
XY: Xiangya hospital
QALYs: Quality-adjusted life-years
ICER: Incremental cost effectiveness ratio
COVID-19: Coronavirus Disease 2019

## References

[1] Schnabel RB, Yin X, Gona P, Larson MG, Beiser AS, McManus DD (2015). 50 year trends in atrial fibrillation prevalence, incidence, risk factors, and mortality in the Framingham heart study: a cohort study. Lancet..

[2] Zhou Z, Hu D (2008). An epidemiological study on the prevalence of atrial fibrillation in the Chinese population of mainland China. J Epidemiol.

[3] Zhang Z, Lei J, Shao X, Dong F, Wang J, Wang D, Wu S, Xie W, Wan J, Chen H, Ji Y, Yi Q, Xu X, Yang Y, Zhai Z, Wang C, Zhang J, Zhang P, Mao Y, Yang X, Xu X, Xia G, Zheng R, Gao Y, Zhu G, Zhu C, Fu Y, Chen H, Yu F, Kuang J, Li Z, Cheng Z, Wu R, Cheng Z, Tong L, Jiang Y, Sun J, Xu Q, Pan H, Wang L, Zeng M, Chen Y, Yu C, Hua J, Tang Y, Ji Y, An J, Zhang Y, Ding Y, Zhang W, Wu X, Chai W, Li J, Yi Q, Wang H, Chen X, Zhang A, Han J, Ying K, Xu X, Shi Z, Sun J, Zhao Q, Liu G, Zhuo J, Shi G, Ding Y, He Z, Lang Z, Hu X, Fan F, Liu H, Sun G, Xing G, Zhang Y, Su G, Ni J, Zhao T, Wang J, Zhang N, Qin S, Huang S, Xu Q, Li Y, Liu Q, Wu Q, Li L, Chen X, Niu Z, Huang J, Zeng D, Yuan Y, Tian Q, Zhang J, Han X, Yang J, Bo B, Huang Y, Luo Q, Pang G, Zheng H, Zhang P, Xu R, Zhang Y, Ni S, Li S, Gong Y, Zhang J, Zhu L, Xia S, Chang Y, Zhang J, Han X, Yuan Y, Tian Q, Zhang H, Xu X, Zhang Y, Pan J, Qin Z, Lao M, Li J, Liu Z, Luo Q, Wang J, Wang N, Yang H, Tang X, Bai X, Chen Y, Han D, Shen S, Jin C, Ye Y, Suo L, Huang X, Wang J, Zhang X, Yang G, Yu G, Zhang S, Yang Y, Cheng J, Duo J, Zhang H, Wang P, Li Y, Wang P, Guo C, Bian T, Cai S, Cheng Z, Wang T, He Y, Huang W, Liu C, Zhao H, Tu F, Zhu Y, Tian G (2019). Trends in hospitalization and in-hospital mortality from VTE, 2007 to 2016, in China. Chest..

[4] Wallentin L, Yusuf S, Ezekowitz MD, Alings M, Flather M, Franzosi MG, Pais P, Dans A, Eikelboom J, Oldgren J, Pogue J, Reilly PA, Yang S, Connolly SJ (2010). Efficacy and safety of dabigatran compared with warfarin at different levels of international normalised ratio control for stroke prevention in atrial fibrillation: an analysis of the RE-LY trial. Lancet..

[5] Sun Y, Hu D, Stevens S, Lokhnygina Y, Becker RC, Berkowitz SD, Breithardt G, Hacke W, Halperin JL, Hankey GJ, Mahaffey KW, Nessel CC, Piccini JP, Singer DE, Fox KAA, Patel MR (2017). Efficacy and safety of rivaroxaban versus warfarin in patients from mainland China with nonvalvular atrial fibrillation: a subgroup analysis from the ROCKET AF trial. Thromb Res.

[6] Chang SS, Wu JH, Liu Y, Zhang T, Du X, Dong JZ (2018). In-hospital direct costs for thromboembolism and bleeding in Chinese patients with atrial fibrillation. Chronic Dis Transl Med.

[7] Young S, Bishop L, Twells L, Dillon C, Hawboldt J, O'Shea P (2011). Comparison of pharmacist managed anticoagulation with usual medical care in a family medicine clinic. BMC Fam Pract.

[8] Nichol MB, Knight TK, Dow T, Wygant G, Borok G, Hauch O, O'Connor R (2008). Quality of anticoagulation monitoring in nonvalvular atrial fibrillation patients: comparison of anticoagulation clinic versus usual care. Ann Pharmacother.

[9] Ansell J, Holden A, Knapic N (1989). Patient self-management of oral anticoagulation guided by capillary (fingerstick) whole blood prothrombin times. Arch Intern Med.

[10] Wells PS, Brown A, Jaffey J, McGahan L, Poon MC, Cimon K (2007). Safety and effectiveness of point-of-care monitoring devices in patients on oral anticoagulant therapy: a meta-analysis. Open Med.

[11] Tuckson RV, Edmunds M, Hodgkins ML (2017). Telehealth. N Engl J Med.

[12] Doraiswamy S, Abraham A, Mamtani R, Cheema S (2020). Use of telehealth during the COVID-19 pandemic: scoping review. J Med Internet Res.

[13] Ryan F, Byrne S, O'Shea S (2009). Randomized controlled trial of supervised patient self-testing of warfarin therapy using an internet-based expert system. J Thromb Haemost.

[14] Comuth WJ, de Maat MPM, van de Kerkhof D, Malczynski J, Husted S, Kristensen SD, Munster AB (2019). Adherence to dabigatran etexilate in atrial fibrillation patients intended to undergo electrical cardioversion. Eur Heart J Cardiovasc Pharmacother.

[15] Zeolla MM, Brodeur MR, Dominelli A, Haines ST, Allie ND (2006). Development and validation of an instrument to determine patient knowledge: the oral anticoagulation knowledge test. Ann Pharmacother.

[16] Pennington M, Grieve R, Sekhon JS, Gregg P, Black N, van der Meulen JH (2013). Cemented, cementless, and hybrid prostheses for total hip replacement: cost effectiveness analysis. BMJ..

[17] Samsa G, Matchar DB, Dolor RJ, Wiklund I, Hedner E, Wygant G, Hauch O, Marple CB, Edwards R (2004). A new instrument for measuring anticoagulation-related quality of life: development and preliminary validation. Health Qual Life Outcomes.

[18] Rosendaal FR, Cannegieter SC, van der Meer FJ, Briet E (1993). A method to determine the optimal intensity of oral anticoagulant therapy. Thromb Haemost.

[19] Brasen CL, Madsen JS, Parkner T, Brandslund I (2019). Home management of warfarin treatment through a real-time supervised telemedicine solution: a randomized controlled trial. Telemed J E Health.

[20] Telenti A, Arvin A, Corey L, Corti D, Diamond MS, Garcia-Sastre A, et al. After the pandemic: perspectives on the future trajectory of COVID-19. Nature. 2021. 10.1038/s41586-021-03792-w. Online ahead of print. 10.1038/s41586-021-03792-w 34237771

[21] Medical AS (2009). Point-of-care international normalized ratio (INR) monitoring devices for patients on long-term oral anticoagulation therapy: an evidence-based analysis. Ont Health Technol Assess Ser.

[22] Matchar DB, Jacobson A, Dolor R, Edson R, Uyeda L, Phibbs CS, Vertrees JE, Shih MC, Holodniy M, Lavori P (2010). Effect of home testing of international normalized ratio on clinical events. N Engl J Med.

[23] Sharma P, Scotland G, Cruickshank M, Tassie E, Fraser C, Burton C, Croal B, Ramsay CR, Brazzelli M (2015). Is self-monitoring an effective option for people receiving long-term vitamin K antagonist therapy? A systematic review and economic evaluation. BMJ Open.

